# Teleconsultation for Children With Developmental Disabilities During the Coronavirus Pandemic: Caregivers’ Experience

**DOI:** 10.7759/cureus.48816

**Published:** 2023-11-14

**Authors:** Urmila Dahake, Jaya Prasad Tripathy, Abhijit Choudhary, Shikha Jain, Akash Bang, Meenakshi Girish

**Affiliations:** 1 Department of Pediatrics, All India Institute of Medical Sciences, Nagpur, Nagpur, IND; 2 Department of Community Medicine, All India Institute of Medical Sciences, Nagpur, Nagpur, IND

**Keywords:** covid-19 pandemic, cerebral palsy (cp), autism, teleconsultation, disability

## Abstract

Background

The unprecedented situation due to the coronavirus disease 2019 (COVID-19) lockdown necessitated the need for teleconsultations with caregivers of children with disabilities. The objective of this study was to explore the acceptability, satisfaction, perceived relevance, and barriers to teleconsultation from a caregiver’s perspective.

Methodology

This was a descriptive qualitative study (telephonic interviews) involving in-depth interviews (IDIs) with the caregivers of children with developmental disabilities who received teleconsultations. Manual content analysis of transcripts of IDIs was done.

Results

Eight IDIs were conducted with the caregivers of children with cerebral palsy, autism, and developmental delay. The respondents expressed increased challenges in managing their children during the pandemic and the need for professional consultation. They also expressed difficulty in accessing professional help during the pandemic due to poor healthcare access and fear of getting COVID-19. The following responses were noted: “For almost a year we couldn’t take her for the therapy,” “We were unable to take him to therapy which resulted in an increase in tightness of his limbs, and he became more irritable.” All respondents preferred video teleconsultations during lockdown due to flexible timings, ease of communication, and no travel restrictions; “I can benefit from teleconsultation because she does not have any physical problem.” However, caregivers of children with physical ailments preferred face-to-face consultation.

Conclusions

Teleconsultation was found to effectively support the treatment and rehabilitation of children with disabilities during the COVID-19 lockdown, although direct face-to-face consultation was preferred by caregivers of children with physical ailments. The use of modern mobile/digital technologies creates new opportunities to improve the quality and accessibility of such services.

## Introduction

According to the World Health Organization, nearly 15-20% of children worldwide have disabilities; 85% of which are in developing countries. As per the Census 2011 in India, nearly 27 million people suffer from various disabilities [1]. There are 8 million children with disabilities in the below 19-year age group, including visual impairment, hearing impairment, speech disorder, movement disorder, intellectual disability, multiple disabilities, and other types of disabilities [1]. Due to body function/intellectual impairment and activity limitation, people with disabilities (PWD) experience participation restriction in routine life situations. This includes access to healthcare services. For PWDs, inequity in accessing healthcare is a global issue, with PWDs having poorer healthcare access which results in poorer health outcomes [1]. Children with disabilities who are dependent on caregivers to access health services face additional challenges. In addition, the absence of transportation to hospitals, unavailability of services within the hospital, attitudinal barriers, discrimination and language barriers, and costs represent critical barriers to access and utilization of healthcare among PWDs [2]. The coronavirus disease 2019 (COVID-19) pandemic has added new dimensions to the challenges faced by children with disabilities and their caregivers. In the wake of the pandemic, a nationwide lockdown was enforced on March 23, 2020, which disrupted public lives. PWDs were disproportionately impacted by the lockdown because of serious disruptions to the services they rely on such as the complete shutdown of public and private transport. This led to an increase in psychological stress among parents/caregivers due to difficulties in managing a child with a disability, especially with behavioral issues, at home in view of a complete ban on recreational activities outside and school closures. The effect of disruption of medical services also added to the woes of the caregivers. This unprecedented situation necessitated the need for teleconsultation with the parents of children with disabilities. Teleconsultation can provide routine clinical guidance by removing access barriers and alleviating stress on the part of the caregiver. Previous applications of telemedicine for children with special healthcare needs have improved access to specialty care by reducing barriers of time and expense associated with travel [3,4]. Parents have found telemedicine consultations to be equally effective as in-person visits and have reported high satisfaction levels [5]. Teleconsultation is suitable for certain disabilities where verbal consultation is sought without the need to examine the child physically. However, teleconsultation involves observing a child on a live screen without actually touching and interacting with the caregivers off-site, which leads to the question, does that suffice? What do the physician and the caregiver feel about this form of consultation compared to face-to-face consultation? These questions need to be answered. The Department of Pediatrics at the All India Institute of Medical Sciences (AIIMS), Nagpur is running a Developmental Disability Clinic on a weekly basis starting from December 2019. Approximately 25 children were regularly being followed up in the clinic till mid-March 2020. However, since March 22, 2020, no child could follow up due to the lockdown situation. Hence, we decided to reach out to the caregivers of these children enrolled with the Developmental Disability Clinic through teleconsultation. There is no evidence in the literature from India on the benefits and challenges of teleconsultation vis-à-vis conventional face-to-face consultation for children with developmental disabilities from a caregiver’s perspective, which we explored through this qualitative inquiry. Due to resource limitations in reaching out to patients with developmental disabilities and their caregivers during this lockdown and the health hazards of doing a face-to-face interview, we planned telephone interviews. The specific objective of this qualitative inquiry was to explore the acceptability, satisfaction, perceived relevance, and barriers to teleconsultation from a caregiver’s perspective.

This article was previously presented as a scientific poster at the 77th Annual Meeting of the American Academy of Cerebral Palsy and Developmental Medicine on September 11, 2023.

## Materials and methods

Study design

This was a descriptive qualitative study involving in-depth interviews (IDIs) with the caregivers of children with developmental disabilities.

Study population

The study population included the caregivers of children with developmental disabilities under regular follow-up at the Developmental Disability clinic at AIIMS, Nagpur. The caregivers were consecutively recruited into the study after obtaining informed consent for receiving teleconsultation.

Data collection

All teleconsultation interviews were conducted from May 2020 to August 2020. The Principal Investigator (PI) conducted teleconsultation sessions with parents of children with disabilities. For conducting these sessions, the following procedure was followed. A validated Google Forms was created for collecting demographic details and information about the child’s disability and current health issues related to the disability discussed during the teleconsultation. A link to book an appointment for a teleconsultation was available at the end of the form. The link to the Google Forms was shared with the caregivers of children with a disability under regular follow-up at the Developmental Disability clinic at AIIMS, Nagpur. The Google Forms also had a consent form to participate in the teleconsultation session and the study. Once the Google Forms was submitted, the PI contacted the caregiver who had consented to the booked appointment date and time The PI conducted the teleconsultation session on the scheduled appointment time. At the end of the teleconsultation session, the PI fixed the time for the IDI.

In-depth interviews

**Figure 1:**
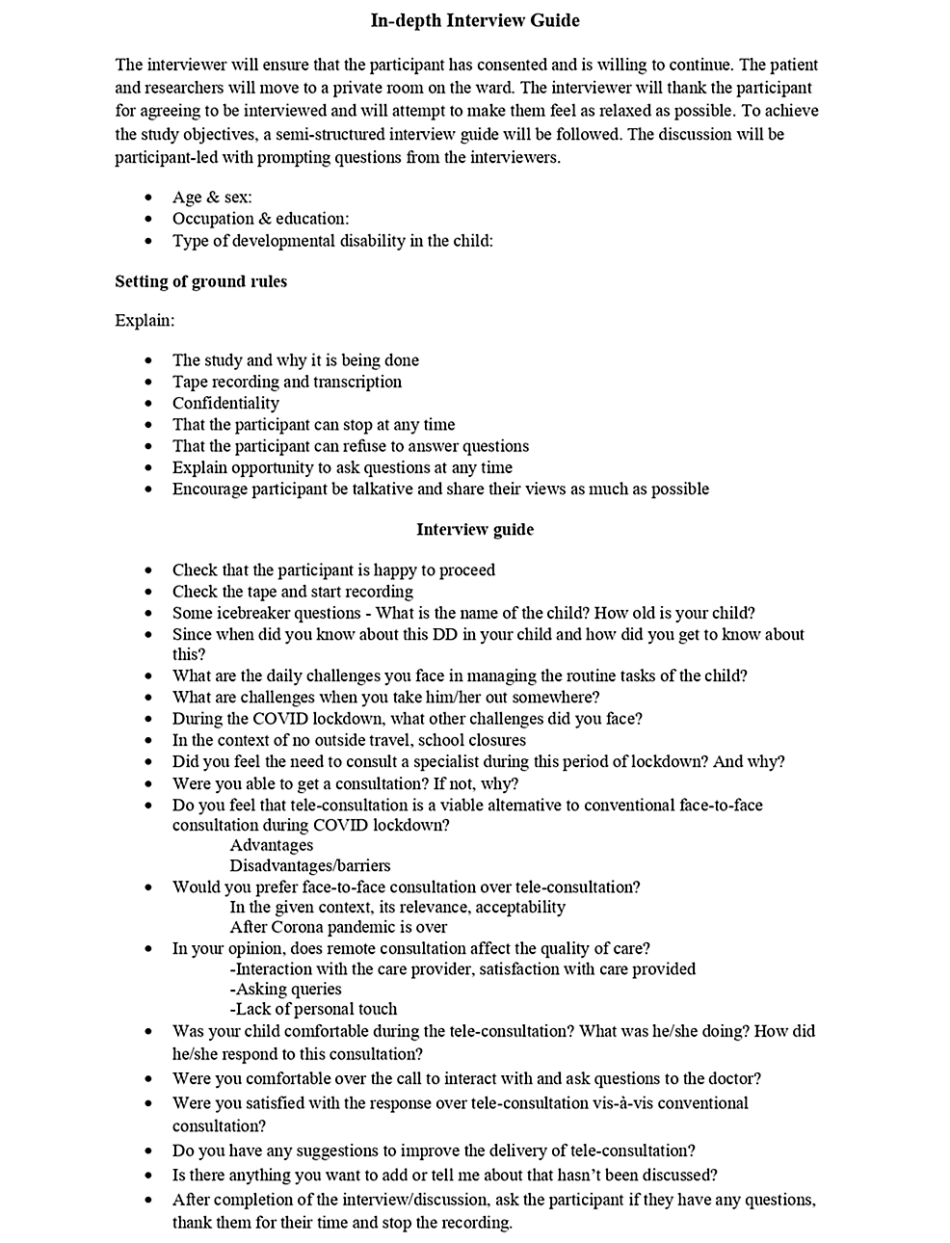
In-depth interview guide.

The PI (MBBS, MD, Developmental Pediatrician and MA in Psychology) and the co-PI (MBBS, MD) who are trained in qualitative research methods conducted the IDIs after obtaining consent from the participants. The IDIs were conducted in the local language (Hindi/Marathi) which both the interviewer and interviewee were comfortable with over the telephone. An interview guide with open-ended questions was used to explore their experiences related to teleconsultation (Figure 1). The interviews were recorded (after obtaining consent). Each interview lasted for around 30-45 minutes. After the interview, the summary of the interviews was read back to the participants to ensure participant validation. As this was a telephonic interview, no incentives were provided to the participants for their participation. The saturation of responses was used to guide the number of interviews.

Qualitative data analysis

The audio-recorded interviews were transcribed manually in the English language by the study investigators as soon as the interviews were over. The transcripts were read multiple times by two investigators (UC and JPT) before coding to increase familiarity with the content. Manual thematic analysis was undertaken to analyze the transcripts [6]. A hierarchical codebook was developed by two study investigators (UC and JPT) by synthesizing codes emerging directly from the transcripts (inductive) and the topic guides (deductive). The initial coding was done independently by the investigators after going through the transcripts. In case of any discrepancy, the codes were discussed and disagreements were resolved. Similar codes were combined to generate themes [7]. Verbatims were presented to support the findings of the caregiver’s experience of the teleconsultation. We adhered to the COREQ guidelines to report the findings of the study [8].

Ethical approval

Ethical and scientific approvals were obtained from the Institutional Ethics Committee and Research Cell of AIIMS, Nagpur. Written informed consent was obtained via Google Forms and verbal consent from the participants was obtained through telephone call before starting the interview. The calls were monitored by an individual not associated with the current research.

## Results

**Figure 2:**
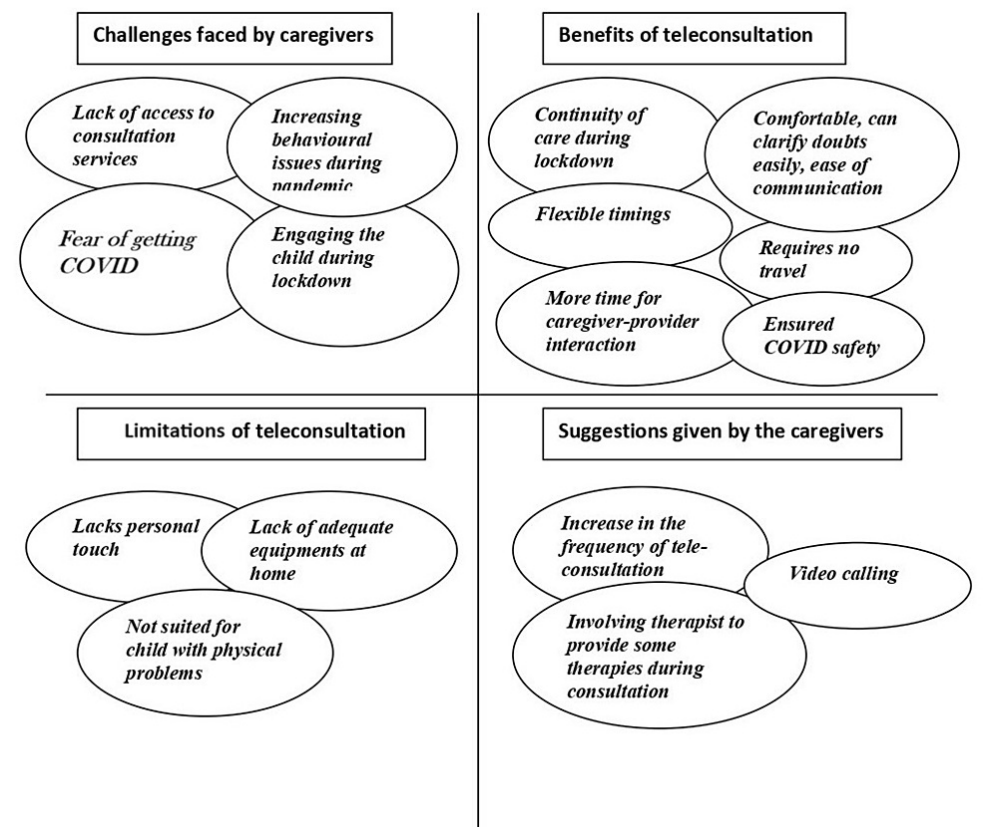
Caregivers’ perspectives on benefits and barriers of teleconsultation.

Eight IDIs were conducted with the caregivers of children with cerebral palsy (two), autism spectrum disorder (three), global developmental delay (two), and specific learning disability (one); half of the children were females (four, 50%). All caregivers were mothers of the children. Analysis of the transcripts yielded the following four major themes: (i) challenges faced by the caregivers, (ii) benefits of teleconsultation, (iii) disadvantages of teleconsultation, and (iv) suggestions given by the caregivers (Figure 2).

Challenges faced by the caregivers

Lack of Access to Consultation Services

Due to the strict COVID-19 lockdown, caregivers could not take their children for counseling and physiotherapy sessions or medical consultations. They were also left wanting in case of medical emergencies.

“For almost a year we couldn’t take her for therapy.”

“Many times we wanted to get a consultation for her. She was having fits but we were not able to reach her neurologist.”

“Everything was closed … consultation was not available outside even if we wanted to.”

Some of the caregivers resorted to therapy sessions at home. However, they did not have the necessary infrastructure such as specific places and toys required for therapy. They were also less confident in managing therapy sessions at home.

Increasing Behavioral Issues During the Pandemic

Almost all caregivers said that the behavioral issues of the child increased during the pandemic as they were confined to their homes during the lockdown.

“Because of lockdown, we could not take him to therapy which resulted in increase in the tightness of his limbs. He became more irritable and difficult to manage.”

“We were not able to take her to the garden also. Her irritability also increased as she was constantly at home. Even her hand movements increased during the lockdown period.”

“Before the pandemic he was attending therapy sessions regularly and we were able to see a positive improvement in him. But after the pandemic as therapy sessions stopped, the changes in him again regressed.”

Engaging the Child During the Lockdown

As the children always remained at home during the lockdown, they became irritable, and keeping them engaged was a big challenge for the caregivers.

“After the lockdown, he is at home only all the time. It is not possible as a parent to keep him engaged all the time and that is very much challenging to us.”

Fear of Getting COVID-19

The fear of contracting COVID-19 infection was also a major concern for caregivers.

“There was opposition from my family because they were worried about us getting COVID-19 if we went outside.”

“I could have visited the hospital … but we were scared of getting in contact with a COVID-19 patient as any hospital would have COVID-19 patients. So this scare was always there in our mind.”

Amid these challenges faced by the caregivers during the pandemic, one caregiver found some positives during lockdown: “Because of the daily travel to therapy sessions, he used to get frequently ill with cough and cold. But now my child is at home I find there is less cough and cold.”

Benefits of teleconsultation

Continuity of Care During Lockdown

All caregivers vocally supported the teleconsultation sessions provided during the pandemic when access to healthcare services was poor. Everyone felt that this is very well-suited for situations such as lockdown. Tele-consultation support came as a big relief to most caregivers, as one of them said, “consultation helped us to think over the issues again and how to deal with it.” Another caregiver said, “I could discuss the issues with a specialist with an expert, sitting comfortably at home.”

Flexible Timings

Caregivers felt that teleconsultation saved a lot of time as it obviates the need for travel and preparation for a hospital visit. On the other hand, frequent direct consultation was difficult for working parents.

“I am getting an appointment whenever I want. So I am quite comfortable.”

“It is challenging for parents to spare time for direct consultation, so it is more helpful for them.”

No Travel Requirements

A major benefit of teleconsultation is that it obviates the need for traveling, thereby saving time, money, and the logistics around traveling. A mother said, “I don’t have leaves in my office and also I need to get somebody’s help as the institute is far. So both parents need to be involved. Even my husband is busy. So for me, it is very useful that I am getting expert advice at home.” Another said, “definitely it is a viable alternative, … teleconsultation has removed the issue of distance or need for travel. Due to this, it is a good option even when things are alright.”

Comfortable, Can Clarify Doubts Easily, and Ease of Communication

Most respondents said that they were very comfortable during the session. Before the consultation, they had written down the doubts on a piece of paper to be discussed during the call. By doing this, they never missed any point. Some of them also expressed that they were more comfortable asking doubts or expressing themselves over an online session compared to a face-to-face consultation.

“Don’t know why in direct consultation I can’t express my doubts that freely … I feel … I can express myself better telephonically.”

More Time for Caregiver-Provider Interaction

The teleconsultation sessions also gave the caregivers enough time to discuss the issues and challenges compared to direct consultations which used to be very brief.

“Any teleconsultation we are getting enough time to discuss, it’s not that we are being asked to finish in 5 minutes.”

Ensured COVID-19 Safety

Teleconsultation also meant that they did not have to step out of their homes and, thus, ensured COVID-19 safety. This was also reported by some of the caregivers.

Teleconsultation After the Pandemic

There was a divided opinion in this regard. Some preferred resorting back to offline consultation, some wanted a mix of both, and others wanted to continue online consultation even after the pandemic was over, mostly those with children having behavioral issues.

“I mainly face issues with her behavioral issues, which I can explain over phone also. Even in the future, I will benefit by telephone consultation only.”

Limitations of teleconsultation

Lacks Personal Touch

All respondents benefited from teleconsultation during the lockdown and supported this initiative. However, some were also of the opinion that teleconsultation cannot replace face-to-face consultation as it lacks a personal touch. However, teleconsultations were certainly looked down upon as a promising alternative to direct consultation in such situations.

“I definitely wish to have an offline consultation, but then I was not able to get it. But I had the option of online consultation.”

“Direct consultation is better as the doctor can actually see the child and we also get satisfaction.”

“If the matter is of superiority, then definitely offline consultations are much superior.”

Not Suited for Children With Physical Problems

Some of the caregivers expressed that children with physical problems require examination by the doctor, and thus, teleconsultation might not be suitable for them. “No I think that direct consultation is better as the doctor can actually see the child and we also get satisfaction.” Video consultation can suffice as an alternative to the direct consultation in case if it is absolutely not available due to lockdown was narrated by one of the caregiver of child with physical disability. “Yes maybe in that case we can go for a video consultation so that we are satisfied that the doctor is able to see our child through the video call.”

On the other hand, children with behavioral problems can be handled well by teleconsultations without being physically examined by a doctor.

“My child does not have any physical problem which needs examination. I mainly face problems with her behavioral issues which can be explained over the phone better than through direct consultation.”

“I can benefit from telephone consultation because she does not have any physical problem.”

Lack of Adequate Equipment at Home

Some of the respondents reported a lack of equipment needed to perform the activities suggested by the expert during the teleconsultation. “Equipment required to perform those activities they are easily available at the therapy center but it is not necessary that it will be available with us too.”

Suggestions given by the caregivers

Most caregivers suggested an increase in the frequency of teleconsultation to daily or thrice weekly. Another caregiver also proposed the involvement of a physiotherapist or speech therapist to provide some therapies during teleconsultation sessions. Video calling was suggested by the caregivers of children with physical disabilities.

## Discussion

This is the first study from India exploring the utility of teleconsultation services for children with developmental disabilities during the pandemic and documenting caregiver’s experiences of teleconsultation vis-à-vis face-to-face consultation. An exploratory study from West Bengal published in 2021 studied challenges in accessing healthcare for adults with disabilities during COVID-19 [9]. The study concluded that there are major challenges that people with disabilities face compared to an average person which worsened during the COVID-19 pandemic. From accessibility to lack of service, from poor financial support to lack of help from society, people with disabilities have to face barriers at every level and survive.

Almost all caregivers felt that teleconsultation was very beneficial during the lockdown to ensure continuity of care, but it cannot build the same rapport and personal connection as direct consultations. This was echoed by previous studies as well [10]. ﻿However, isolated examples of particularly strong rapport-building in teleconsultations were also experienced in our study, suggesting that this may be amenable to training.

The caregivers felt that there was enough time for interaction during the teleconsultation sessions compared to direct consultation which is generally “very rushed.” However, previous studies comparing both modes of consultation have reported more time being spent in direct consultations [10]. These differences could be due to varied settings. The present study was done in a middle-income setting with a high patient load, thereby reducing the time for direct consultation.

Caregivers of children with physical disabilities preferred direct consultation compared to teleconsultation compared to caregivers of children with autism spectrum disorder who preferred teleconsultation. Similar results were obtained by Corona et al. where the authors studied 115 families with autism spectrum disorder [11]. They participated in a behavioral intervention and support service model either in-person, through telemedicine, or through a hybrid service model. Some of the caregivers believed that children with behavioral issues could be managed much better through online consultations, whereas those with physical problems or severe issues need direct consultation.

“Used appropriately, telemedicine can be an important resource for health systems, but also a connecting link between the public healthcare services that will be reconfigured after the COVID-19 pandemic crisis,” according to Igor Codreanu, Health Program Coordinator at UNICEF Moldova [12]. An opinion published by Provenzi et al. suggested that telemedicine strategies should not be considered as an emergency response [13]. A family-centered telemedicine program, namely, the Engaging with Families in On-line Rehabilitation of Children during the Epidemic (EnFORCE) program, was launched by the authors and was well-received by parents. A recent survey conducted in the biennium 2018-2019 by the Italian National Institute of Statistics (ISTAT) revealed that approximately 33% of families had no computer or tablet at home; this estimate was lower (14%) for families with at least one child [14]. Only 22% of families had a one-to-one member-device ratio, and families with low socioeconomic status were especially lacking the availability of computers and tablets [15].

With the growing digitalization of healthcare, the availability of technological devices in home environments becomes a key requirement for accessing healthcare services [16].

The study has the following strengths. First, the study was able to provide in-depth insights into the utility of teleconsultation from a caregiver’s perspective during the pandemic-related lockdown. Second, we followed the COREQ guidelines for reporting the findings from this qualitative inquiry [8].

However, there were some limitations in this study. First, only eight caregivers of children with developmental disabilities were interviewed in this study. This might limit the external generalizability of the findings. However, the richness of the interviews provided a useful understanding of this complex behavior. Second, the doctor may have altered his/her consulting during teleconsultation sessions.

## Conclusions

This study demonstrated the suitability and satisfaction of teleconsultation as an alternative to face-to-face clinic appointments, especially during lockdowns. Caring for children with developmental disabilities presented many complexities for caregivers during the lockdown due to lack of access to medical care and restricted mobility. Teleconsultation ensured continuity of care during the lockdown. However, one must remember that virtual consultations cannot build the same rapport and connection with patients that are gained from face-to-face meetings and should not be used as a complete substitute for the latter.
